# An Expanded Wing Crack Model for Fracture and Mechanical Behavior of Sandstone Under Triaxial Compression

**DOI:** 10.3390/ma17235973

**Published:** 2024-12-06

**Authors:** Esraa Alomari, Kam Ng, Lokendra Khatri

**Affiliations:** Civil and Architectural Engineering and Construction Management, University of Wyoming, Laramie, WY 82071, USA; ealomari@uwyo.edu (E.A.); lokendra.khatri320@gmail.com (L.K.)

**Keywords:** failure model, confining pressure, wing crack model, normalized critical crack length, type-I fracture toughness

## Abstract

A new model is developed to predict the mechanical behavior of brittle sandstone under triaxial compression. The proposed model aims to determine the normalized critical crack length (*L*_*cr*_), through which the failure strength (*σ*_*f*_) of sandstone can be estimated based on fracture mechanics applied to secondary cracks emanating from pre-existing flaws, while considering the interaction of neighboring cracks. In this study, the wing crack model developed by Ashby and Hallam (1986) was adopted to account for the total stress intensity at the crack tip (*K*_*I*_) as the summation of the stress intensity due to crack initiation and crack interaction. The proposed model is developed by first deriving the *L*_*cr*_ and then setting the crack length equal to the *L*_*cr*_. Next, the total stress intensity is set equal to the rock fracture toughness in the original equation of *K*_*I*_, resulting in an estimate of the *σ*_*f*_. Finally, to evaluate the performance of the proposed model on predicting *σ*_*f*_, theoretical results are compared with laboratory data obtained on sandstone formations collected from Wyoming and the published literature. Moreover, the *σ*_*f*_ predicted by our proposed model is compared with those predicted from other failure criteria from the literature. The comparison shows that the proposed model better predicts the rock failure strength under triaxial compression, based on the lowest RMSE and MAD values of 36.95 and 30.93, respectively.

## 1. Introduction

Brittle fracture in compression is a complex behavior that has attracted many past research developments on different failure criteria to describe fracture mechanisms [1]. Various research has proposed different approaches to model the brittle fracture of rocks under compression. Although some analytical models agree quite well with measured experimental data, the main contribution of most experimental studies is limited to uniaxial or biaxial compression tests conducted on pre-cracked artificial specimens [2]. Further research investigations are needed to develop reliable analytical models for describing the mechanical behavior of natural rock specimens under triaxial compression [3,4,5].

Modeling the brittle failure of rocks is one of the greatest challenges due to the heterogeneous nature of rocks containing pores, flaws, and other weaknesses [6,7,8]. Furthermore, such discontinuities play a significant role in the initiation and propagation of new cracks [9,10], which affect their strength behaviors. In a study by Lu and Yan of the concrete fracturing behavior (which represents brittle materials behavior such as rocks) fracture, they investigated the effect of the chevron notch inclination on the failure strength. The study indicated that the maximum failure load increased with increasing the inclination angle of the notch. Furthermore, the fracture propagation angle also increased with the notch inclination angle, and wing fractures were observed [11]. However, it is difficult to trace crack initiation and propagation during experiments due to the non-transparency nature of the rock.

Advanced measurement techniques such as acoustic emissions (AE) detection [12,13], computerized tomography (CT) scanning system [14], and scanning electron microscope (SEM) [15,16] have been applied to monitor the fracture process. However, the AE signals can be affected by various factors such as noise, attenuation, reflection, and sample geometry. CT is too expensive and time-consuming, and the SEM cannot capture the image of wet samples as they may be damaged by the vacuum required during operation.

A fracture mechanism due to pre-existing microcracks was proposed by Schulson et al. [17]. Unlike the pore-emanated cracking model for brittle failure of sedimentary rocks under triaxial compression [18], this fracture mechanism is concerned with wing cracks growing from an initial inclined flaw. In addition to wing crack initiation at the tip of preexisting flaws, they considered the effect of additional cracks emanating adjacent to wing cracks (splay cracks). The model states that the failure of rock mass is assumed to be initiated by the bending of microcolumns formed by splay and wing cracks, and the applied stress to trigger faulting under uniaxial loading is expressed in terms of the type-I fracture toughness ($K_{IC}$), coefficient of friction (*μ*), slenderness ratio of the microcolumns, and the angle between the direction of the principal stress and wing crack. However, this model underestimated the failure stress due to considering a single microcolumn for crack propagation and neglecting the frictional resistance gained by adjacent columns.

Paliwal and Ramesh [19] proposed a micro-mechanical damage model for brittle failure under uniaxial compression based on frictional sliding of pre-existing flaws. As a result of compressive loading, wing cracks are nucleated at the flaw tips, in addition to interactions among the growing cracks, which give an effective stress field around the crack. The model describes that the compressive strength increases with the increases in mean flaw size and the μ. However, the triaxial compression condition was not included in the study in addition to the several assumptions made in developing the model due to the lack of experimental data. Zhang et al. [20] have developed a modified sliding crack model to estimate the stress intensity factor (SIF) and the crack length (*l*) under the effect of external stresses. The SIF has been derived in the context of Linear Elastic Fracture Mechanics (LEFM) and weight function. The formula of SIF shows that the sliding crack is highly influenced by lateral stress. However, a functional relationship between the crack length and principal stresses (i.e., $\sigma_{1}$ and $\sigma_{3}$) was obtained without a quantitative estimation of the failure strength.

Hoek and Brown [21] have proposed an empirical failure criterion that provides practical means to estimate the rock triaxial strength using experimental data from triaxial compression tests. This failure criterion is adequate and easily applied for strength prediction. However, the criterion application requires a measured uniaxial compressive strength (UCS) of the rock to estimate the triaxial compressive strength. Another common failure criterion is the Mohr–Coulomb (MC) criterion. The MC criterion assumes that failure occurs at a particular combination of the maximum and minimum principal stresses. However, this criterion requires conducting several triaxial compression tests in the calculation of the shear strength parameters (i.e., friction angle and cohesion) before applying the failure model and estimating the failure strength.

Conventional triaxial compression tests can be employed to obtain the parameters of failure criteria to define a rock strength for relevant rock engineering projects. However, they are time-consuming and expensive for testing many samples, and therefore, not readily available at an early stage of a project. Congruent to the above-mentioned limitations, there is an interest in developing an analytical model that generally provides a good prediction of rock triaxial compressive strength based on easily measured or available rock properties.

This research is motivated by the fact that fracture of brittle materials is often induced by the growth and propagation of wing-like flaws based on a micromechanical damage criterion originally formulated by Ashby and Hallam [22]. The total stress intensity at the crack tip $K_{I}$ was adopted from the original wing crack model, and an expanded analytical model was proposed for the normalized critical crack length ($L_{cr}$), which is substituted in the $K_{I}$ equation to estimate the corresponding failure strength. The proposed model enables a systematical study of the effect of the *μ*, stress ratio (λ), and *K*_*I*C_ on the failure strength ($\sigma_{f}$) under a triaxial compression condition. This study also investigates the sensitivity of the critical crack length (i.e., the crack length at the unstable growth stage) to the initial damage level of the rock and confining pressure. The capability of the proposed model in estimating compressive strength is compared with those of other failure criteria that are commonly employed in rock engineering for an extensive database of triaxial compression tests conducted on sandstone samples collected from Wyoming and published data from the literature for different sandstone formations.

## 2. Materials and Methods

### 2.1. Wing Crack Model

Ashby and Hallam [22] have developed a wing crack model for the growth and interaction of cracks in brittle solids under compression. Consider a plate containing a pre-existing crack of length 2a subjected to major and minor principal stresses of $\sigma_{1}$ and $\sigma_{3}$, respectively, the crack is inclined with an angle of *Ψ* from the major principal stress $\sigma_{1}$ direction. The sign convention states that stresses are considered positive if tensile and negative if compressive. The shear stress tends to make the cracks slide but the frictional stress opposes the sliding (i.e., the coefficient of friction μ). The field is predominantly shear on the plane of the initial cracks. However, for the planes that lie at an angle from the crack tip, a normal stress appears that tends to cause type-I wing cracks. Therefore, the type-I fracture toughness is found by seeking the plane of the maximum tensile stress.

The wing crack model attributes the rock brittle failure to tensile cracks that emanate from the tips of pre-existing microcracks. The wing cracks initiate at an angle of 45° with respect to the pre-existing crack tip, where the tensile stress is maximum. The model states that the stress required for crack initiation depends on the initial crack half-length (a) and orientation, the μ, and the stress state. Once wing cracks are initiated, and as stress increases, further sliding of the main crack causes them to grow further parallel to the applied maximum stress [22]. The stress intensity ($K_{I}$) due to cracks initiation is given by the following equation:

(1) $$K_{I}=\frac{\sigma_{1}\sqrt{\pi a}}{\sqrt{3}}\;\times \left\{\left(1-\lambda \right){\left(1+\mu^{2}\right)}^{1/2}-\left(1+\lambda \right)\;\mu \right\}$$

where a is the half-length of the pre-existing crack, λ is the stress ratio ($\sigma_{3}/\sigma_{1}$), and μ is defined as $tan\left(\varphi \right)$. With the increase in stress, these cracks begin to interact and extend further. Extension of wing cracks in an array of micro-cracks divides the solid into slender micro columns. This allows the resultant forces to be treated as shear forces, axial forces, and bending moments. Based on a comprehensive semi-circular bend test on concrete samples, Lu and Yan [12] found that the type-I crack mode is only guaranteed when the crack initiation angle (*Ψ*) is less than 30° or when the theoretical angle is over 70° in the triaxial case. The mixed type-I and type-II failure modes will be experienced when the *Ψ* angle falls between 45° and 70°, and the pure type-II mode is guaranteed when the *Ψ* angle exceeds 70.53°. Hence, it is important to note that the proposed wing crack model derived in Section 2.2 based on $K_{I}$ for type-I mode is mostly applicable to rocks with *Ψ* angles less than 30°.

The governing equation for the stress intensity factor ($K_{I}^{I}$) due to microcracks interaction is presented in Equation (2). The term outside the curly brackets is the crack-opening stress intensity caused by the major principal stress $\sigma_{1}$, while the term inside represents the crack-closing stress intensity caused by the minor principal stress $\sigma_{3}$ based on λ

(2) $$K_{I}^{I}=\frac{\sqrt{2D\times \left(L+\alpha \right)\times \pi a}}{\pi }\sigma_{1}\times \sqrt{\left\{\left[1-\frac{8}{\pi }\;D\;\lambda \;{\left(L+\alpha \right)}^{3}\right]\left[1-\frac{2}{\pi }\;D\;\lambda \;{\left(L+\alpha \right)}^{3}\right]\right\}}$$

where $\alpha =cos\left(\Psi \right)=\frac{1}{\sqrt{2}}\;$since the angle Ψ under the maximum tensile stress is $45^{{}^{\circ}}$; therefore, α = 0.71, and D measures the initial level of damage and equals $\pi a^{2}N_{A}$, where $N_{A}$ represents the number of initial cracks per unit area.

### 2.2. Derivation of the Proposed Model

Short cracks do not often interact, and the stress intensity at the crack tip is the same as that of an isolated crack given by Equation (1). However, as the cracks grow, they start to interact by mechanisms involving bending and buckling which increase the stress intensity at the crack tip [23]. The total stress intensity ($K_{I}$) at the crack tip given by Equation (3) below is derived by adding the stress contributions from external compression loading, given by Equation (1), and crack interaction, given by Equation (2):

(3) $$K_{I}=\frac{\sigma_{1}\sqrt{\pi a}}{\sqrt{3}}\;\times \left\{\left(1-\lambda \right){\left(1+\mu^{2}\right)}^{1/2}-\left(1+\lambda \right)\;\mu \right\}+\frac{\sqrt{2\;D_{o}\times \left(L+\alpha \right)\times \pi a}}{\pi }\sigma_{1}\times \sqrt{\left\{\left[1-\frac{8}{\pi }D_{o}\;\lambda \;{\left(L+\alpha \right)}^{3}\right]\left[1-\frac{2}{\pi }D_{o}\;\lambda \;{\left(L+\alpha \right)}^{3}\right]\right\}}$$

The brittle deformation of rocks is predominantly governed by the growth and coalescence of cracks [24]. The higher the stress intensity at the crack tip, the easier the wing cracks grow. Increasing the major principal stress $\sigma_{1}$ will increase the stress intensity at the crack tip, which promotes crack propagation. However, confinement decreases the stress intensity at the crack tip and inhibits crack propagation [25]. The maximum growth length of the crack before failure is known as the critical crack length, which is reached due to crack interaction as crack growth is always stable under triaxial compression. The stress intensity ($K_{I}^{I}$) caused by cracks interaction, given by Equation (2), is differentiated with respect to crack length L as $\frac{dK_{I}^{I}}{dL}$, given by Equation (4):

(4) $$\;\frac{\mathrm{dK}}{\mathrm{dL}}=\frac{0.029\;{\left(\sigma_{1\;}\sqrt{\pi a}\right)}^{2}\times \left\{\left(L+\alpha \right)-0.48\;\lambda \;{\left(L+\alpha \right)}^{4}+0.038\;\lambda^{2}{\left(L+\alpha \right)}^{7}\right\}}{2k}$$

Setting Equation (4) to zero leads to the following:

(5) $$0=0.2\left(L+0.71\right)-0.65\lambda D{\left(L+0.71\right)}^{4}+0.33\lambda^{2}D^{2}{\left(L+0.71\right)}^{7}$$

Solving Equation (5) using algebra, the L =$\;L_{cr}$ can be expressed in terms of *D* and *λ* by the following equation:

(6) $$L\mathrm{cr}=\sqrt[{3}]{\frac{0.382}{\lambda D}}-0.71$$

The *L_cr_* obtained from Equation (6) indicates the unstable growth regime of cracks emanating from flaw tips. Replacing L in Equation (3) with the Lcr determined from Equation (6) and setting $K_{I}=K_{IC}$, a new model describing the rock failure that accounts for the triaxial condition is proposed as given by Equation (7). This proposed model predicts the λ and therefore the failure strength $\sigma_{f}$.

(7) $$\begin{array}{c} \frac{k_{\mathrm{Ic}}\lambda }{\sigma_{3}\sqrt{\pi a}}=\frac{1}{\sqrt{3}}\times \left\{\left(1-\lambda \right){\left(1+\mu^{2}\right)}^{1/2}-\left(1+\lambda \right)\mu \right\}+\frac{\sqrt{2D\times \left(\left(\sqrt[{3}]{\frac{0.382}{\lambda D}}-0.71\right)+\alpha \right)}}{\pi }\\ \times \sqrt{\left\{\left[1-\frac{8}{\pi }D\lambda {\left(\left(\sqrt[{3}]{\frac{0.382}{\lambda D}}-0.71\right)+\alpha \right)}^{3}\right]\left[1-\frac{2}{\pi }D\lambda {\left(\left(\sqrt[{3}]{\frac{0.382}{\lambda D}}-0.71\right)+\alpha \right)}^{3}\right]\right\}} \end{array}$$

Substituting the α=0.71 [21], Equation (7) is simplified to Equation (8):

(8) $$\frac{k_{\mathrm{Ic}}\lambda }{\sigma_{3}\sqrt{\pi a}}=\frac{1}{\sqrt{3}}\times \left\{\left(1-\lambda \right){\left(1+\mu^{2}\right)}^{1/2}-\left(1+\lambda \right)\mu \right\}+\frac{\sqrt{2D\times \left(\sqrt[{3}]{\frac{0.382}{\lambda D}}\right)}}{\pi }\times \sqrt{\left\{\left[1-\frac{8}{\pi }D\lambda \times \frac{0.382}{\lambda D}\right]\left[1-\frac{2}{\pi }D\lambda \times \frac{0.382}{\lambda D}\right]\right\}}$$

Further simplifying Equation (8) results in the following:

(9) $$\frac{k_{\mathrm{Ic}}\lambda }{\sigma_{3}\sqrt{\pi a}}=\frac{1}{\sqrt{3}}\times \left\{\left(1-\lambda \right){\left(1+\mu^{2}\right)}^{1/2}-\left(1+\lambda \right)\mu \right\}+\frac{\sqrt{2D\times \left(\sqrt[{3}]{\frac{0.382}{\lambda D}}\right)}}{\pi }\times \sqrt{\left\{1-3.19D\lambda \frac{0.382}{\lambda D}+1.632\lambda^{2}D^{2}{\frac{0.382}{\lambda D}}^{2}\right\}}$$

Rearranging Equation (9) leads to the following:

(10) $$\frac{k_{Ic}\lambda }{\sigma_{3}\sqrt{\pi a}}=\frac{1}{\sqrt{3}}\left(\left(1-\lambda \right){\left(1+\mu^{2}\right)}^{1/2}-\left(1+\lambda \right)\mu \right)+\frac{0.927\sqrt{D}}{\pi }\sqrt[{6}]{\frac{0.382}{\lambda D}}$$

Solving the λ value by equating the two sides of Equation (10), the maximum stress $\sigma_{1}$ can be determined according to the corresponding confining pressure since $\lambda =\sigma_{3}/\sigma_{1}$.

### 2.3. Determination of the Model Parameters

For a practical application of the proposed model given by Equation (10), we will need the model parameters: *a*, $K_{Ic}$, *D*, and μ. The $K_{Ic}$ is assumed to be 1.27 MPa$\sqrt{m}$, based on experimental results of type-I fracture toughness tests conducted on 34 sandstone samples collected from three wells drilled in Piceance Basin, Garfield County, Colorado using short-rod specimens [26]. The μ is calculated as tanφ, where φ is the internal friction angle of the rock, or the average μ can be generally assumed as 0.55, based on experimental measurements of friction angle for different sandstones [27].

For the determination of *a*, the basic form of crack initiation in confined rocks appears in the literature with the form given by Equation (11):

(11) $$\sigma_{1}=c_{1}\;\sigma_{3}-\sigma_{o}$$

where $c_{1}$ and $\sigma_{o}$ are material properties, and $\sigma_{1}$ and $\sigma_{3}$ are the axial stress and confining pressure, respectively. According to Nemat-Nasser and Horri [28] and Ashby and Hallam [22], cracks initiate when the stress intensity at the crack tip $K_{I}$ reaches the $K_{IC}$ of the rock. The stress $\left(\sigma_{1}\right)$ at cracks initiation is detected experimentally by the start of Acoustic Emissions (AE), the first non-linearity of the stress–strain curve, or by the dilation of the sample [28] and can be calculated according to Equation (12):

(12) $$\sigma_{1}=\frac{{\left(1+\mu^{2}\right)}^{1/2}+\mu }{{\left(1+\mu^{2}\right)}^{1/2}-\mu }\;\sigma_{3}-\left(\frac{\sqrt{3}}{{\left(1+\mu^{2}\right)}^{1/2}-\mu }\right)\left(\frac{K_{IC\;}}{\sqrt{\pi a}}\right)$$

By fitting the linear relationship between confining pressure and the axial crack initiation stress represented in Equation (12) with experimental data of crack initiation detected by the start of acoustic emissions, the parameter *a* can be determined [27]. Experimental data of acoustic emissions for crack initiation is plotted on the axis of $\sigma_{1}$ and $\sigma_{3}$ with $\sigma_{1}$ representing the *Y*-axis and $\sigma_{3}$ representing the *X*-axis to allow for comparison with the crack initiation criterion to give (*a*) as the intercept of the linear relationship. Results of crack initiation of triaxial compression experiments conducted on medium-grained Buntsandstone by Gowd and Rummel [27] showed that 2*a* is 2 mm.

For the determination of *D*, Ashby and Sammis [29] developed a microcrack damage evolution model in brittle solids based on the initiation and propagation of microfractures from preexisting cracks and described the evolution of damage with strain. The same experimental data are fitted in the damage model proposed by Ashby and Sammis [29] to produce a *D* of 0.15. This value is based on a specific sandstone formation and may not be representative of all sandstones; however, a parametric study is presented in Section 4 of this paper to show how sensitive the $L_{cr}$ is to the *D*.

Substituting the constants of the proposed model (i.e., $K_{Ic}$, *a*, μ, and *D*), the *λ* can be calculated from Equation (10), through which the failure strength $\sigma_{f}\;$can be determined under different $\sigma_{3}$. By adopting the proposed model, no experimental tests are required to determine the model parameters in the prediction of the rock failure strength.

### 2.4. Validation of the Proposed Model

#### 2.4.1. Triaxial Test Data on Sandstone from Wyoming

**Figure 1:**
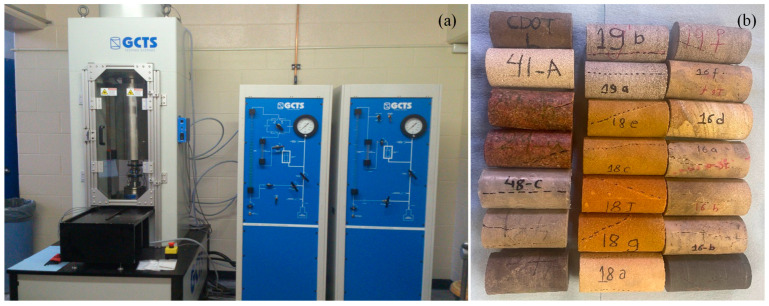
(**a**) Triaxial testing system at the University of Wyoming, and (**b**) Tested specimen.

To evaluate the performance of the proposed model in predicting rock compressive strength, an extensive database of triaxial compression test results on different sandstone formations obtained from our experimental program and collected from published literature was developed. The failure of compressive strengths $\sigma_{f}$ estimated using the proposed model are compared with the corresponding measured $\sigma_{f}$ from triaxial experiments. Sandstone cores collected from Wyoming, USA were used for a series of triaxial compressive tests. The dry density of the rock specimens varies from 2.153 to 2.659 g/cm^3^. Macroscale defects on specimens were not observed before testing [30]. ASTM D7012 (2014) standard [31] was adopted for the triaxial compression tests at the engineering laboratory at the University of Wyoming as shown in Figure 1a. Tested specimens after failure are shown in Figure 1b.

**Figure 2:**
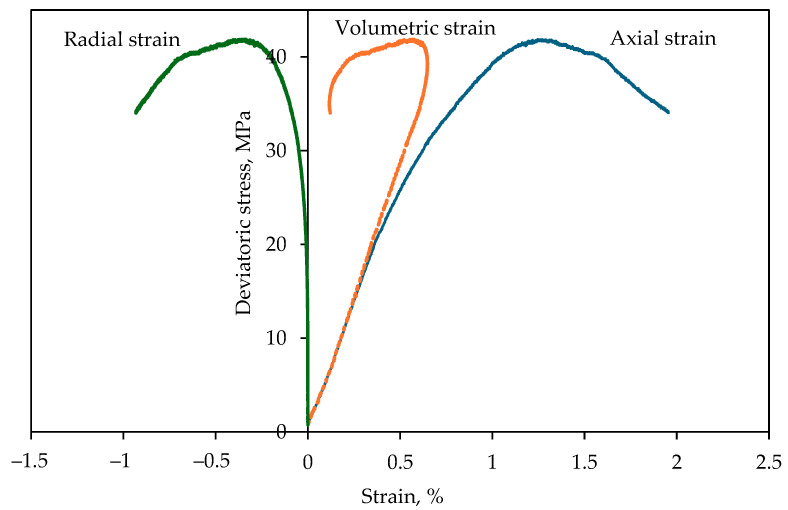
Axial, radial, and volumetric strain curves for Arikaree sandstone.

Each triaxial test was conducted in two stages. In the first stage, a target confining pressure was increased at a rate of 5 MPa/min. In the second stage, deviatoric stress was applied until the specimen failed or reached a 5% axial strain limit. Before testing, Linear Variable Differential Transformers (LVDTs) were instrumented on each specimen to measure the axial and radial LVDT to measure the radial strain. Figure 2 shows an example of a stress–strain plot of Arikaree Sandstone showing the axial, volumetric, and radial strain curves.

The sandstone formation, geological age, core depth, physical properties, and strength properties of the tested rock samples from Wyoming are summarized in Table 1. The samples were tested under confinements ranging from 4 to 10 MPa since they were collected from shallow depths and have a compressive strength ranging from 14.00 to 143.53 MPa.

#### 2.4.2. Triaxial Test Data on Sandstone from the Published Literature

Experimental data of different sandstones under triaxial compression were collected from the literature and used in this validation study. Nine different sandstone formations collected from the surface and various depths and have porosities ranging from 1.5 to 23% were included in this study to create a wide representative dataset in order to better evaluate the performance of the proposed model on predicting the failure strength $\sigma_{f}$. These sandstones have porosities ranging from 1.5 to 23% and were tested under confinements ranging from 3 to 60 MPa with compressive strengths ranging from 58 to 241.81 MPa. Table 2 summarizes the formation, porosity, and strength properties of sandstones from the literature.

## 3. Discussion

Four failure criteria that have gained wide acceptance in rock engineering are selected for comparison with the proposed model in the prediction of the compressive strength of sandstone. The four criteria are the Hoek–Brown criterion, Griffith criterion, Renshaw and Schulson criterion, and Wiebols and Cook criterion. Hoek–Brown and Griffith’s criteria are widely accepted criteria for rock engineering. Renshaw and Schulson’s criterion was expanded from the wing crack model, which is similar to our proposed model, except that they take into consideration the slenderness ratio of the secondary crack rather than the density of preexisting cracks. The Wiebols and Cook criterion requires measured parameters, such as the internal friction angle and UCS for the determination of the failure strength. The main difference between our proposed model and the selected failure criteria is the derivation of the failure strength, as each criterion includes different parameters to estimate $\sigma_{1}$.

**Figure 3:**
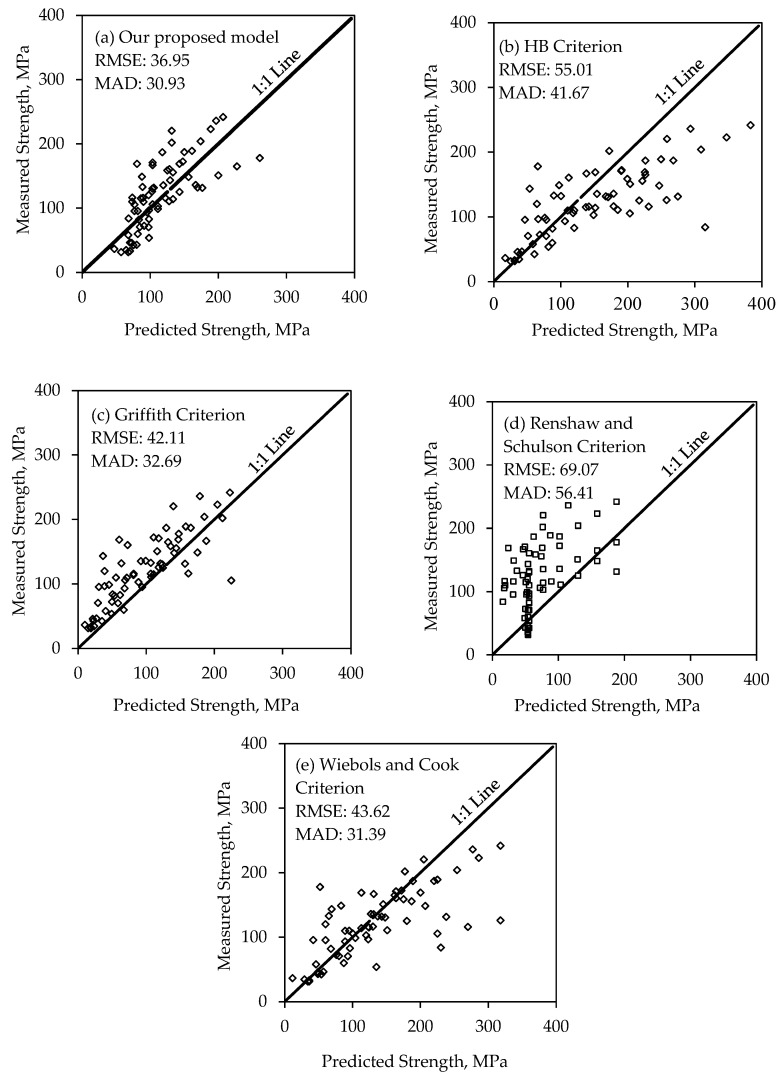
Measured strength versus predicted for (**a**) our proposed model, (**b**) HB criterion, (**c**) Griffith criterion, (**d**) Renshaw and Schulson criterion, and (**e**) Wiebols and Cook criterion.

The performances of the proposed model and four failure criteria in predicting $\sigma_{f}$ are evaluated based on the RMSE and MAD values determined from Equations (13) and (14), respectively, where $y_{\left(i\right)}$ is the measured failure strength, ${\hat{y}}_{\left(i\right)}$ is the predicted failure strength, and *num* is the number of data points. It is desirable to have a small RMSE and MAD for a reasonable candidate model. The RSME and MAD values are inserted in Figure 3 for comparison. In addition, their performances are evaluated based on the data scatteredness along the 1:1 lines of the plots comparing measured to predicted $\sigma_{1}$ in Figure 3. A total of 67 triaxial compression test data on sandstones were used to evaluate the performances of the proposed model and the other four failure criteria. Among the 67 data, 46 triaxial sandstone data were collected from literature and 21 triaxial data were determined for sandstones from Wyoming.

(13) $$\mathrm{RMSE}=\sqrt{\frac{\sum {\left(y_{\left(i\right)}-{\hat{y}}_{\left(i\right)}\right)}^{2}}{\mathrm{num}}}$$

(14) $$\mathrm{MAD}=\frac{\sum \left|y_{\left(i\right)}-{\hat{y}}_{\left(i\right)}\right|}{\mathrm{num}}$$

### 3.1. Our Proposed Model

Based on the statistical results of predicted strength, our proposed model gives the best prediction of $\sigma_{f}$ based on the lowest RMSE and MAD values of 36.95 and 30.93, respectively, with 52% of the data points above the 1:1 line and 48% of the data points under the 1:1 line, as shown in Figure 3a, indicating that the model slightly overestimates the compressive strength $\sigma_{f}$. This is attributed to the moderate to high range of confining pressures under which the rocks are tested (i.e., $\sigma_{3}\sim$20–60 MPa) that results in higher measured to predicted strength ratio and therefore, overestimated $\sigma_{f}$. The mean measured to the predicted ratio equals 1.06, indicating a good estimation of the $\sigma_{f}$.

**Figure 4:**
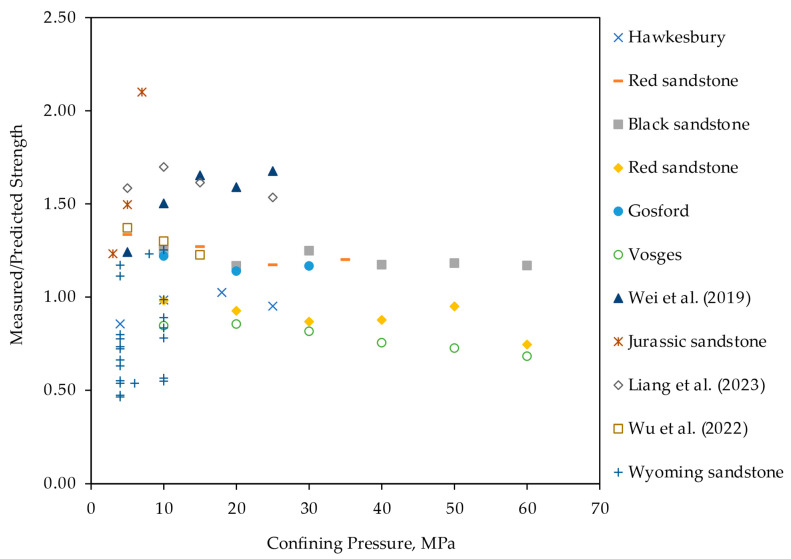
Measured to predicted strength ratio versus confining pressure [37,38,39].

Figure 4 shows the ratio of measured to predicted ratio versus the confining pressure for all sandstones from Wyoming and the literature. The measured-to-predicted ratio decreases with the increase in confining pressure, indicating that higher confinements result in an overestimation of the predicted $\sigma_{f}$ using Equation (10) that indicates higher failure strength under a higher stress ratio λ. For Wyoming sandstones collected from surface and shallow depths and tested at lower confining pressures ranging from 4 to 10 MPa, the strength ratios are mostly lower than 1.0, indicating the overprediction of $\sigma_{f}$ from the proposed model. Although these sandstones were tested under relatively low confinements, they have high internal friction angles (i.e., Average $\varphi =45^{{}^{\circ}}$). This explains the higher predicted $\sigma_{1}\;$due to the proportional relationship between the failure strength and the friction coefficient according to Equation (10).

### 3.2. Hoek Brown Criterion

The Hoek–Brown (HB) criterion is widely accepted for estimating the strength of jointed rock masses [21]. The Hoek and Brown criterion is developed empirically by fitting the results of triaxial tests conducted on intact rock samples and used to describe the nonlinear increase in the peak strength with confinement. The HB criterion for intact rock is expressed in terms of major and minor principal stresses ($\sigma_{1}$ and $\sigma_{3}$), respectively, by Equation (15):

(15) $$\sigma_{1}=\sigma_{3}+\sigma_{ci}\;\sqrt{m_{i}\;\frac{\sigma_{3}}{\sigma_{ci}}+1}$$

where $\sigma_{ci}$ is the rock UCS, and $m_{i}$ is a material constant for intact rock. Letting $m_{i}\;$= 17 for sandstone [40] and using test data from one uniaxial and triaxial compression tests, the failure strength $\sigma_{f}$ can be predicted using this criterion. The HB criterion overpredicts the $\sigma_{f}$ under high confinements (i.e., $\sigma_{3}$ > 40 MPa), as shown in Figure 3b and as seen in the case of Black and Red sandstones [41]. As shown in Figure 3b, the HB criterion overpredicts 76% of the $\sigma_{f}$ data with having higher predicted strength than the measured, especially at higher confinements, according to Equation (15), which states that the failure strength is proportional to the confining pressure; therefore, higher confinement results in higher calculated strength.

Due to the adequacy of strength prediction and the convenient application of this criterion, the material constants are determined empirically and there is no fundamental relationship between them and the rock’s physical properties [42]. Moreover, the HB criterion requires the estimation or measurement of the rock UCS, which is not required in the proposed model.

### 3.3. Griffith Criterion

Griffith theory is one of the most significant advances in rock fracture mechanisms that is physically plausible and developed to derive a failure criterion under compressive stresses [43]. The Griffith criterion has been applied to triaxial compression tests in the form of the following:

(16) $${\left(\sigma_{1}-\sigma_{3}\right)}^{2}-8\;T_{o}\;\left(\sigma_{1}+\sigma_{3}\right)=0$$

where $T_{o}$ is the uniaxial tensile strength or $\sigma_{t}$. According to the original Griffith theory, the rock’s compressive strength is eight times the tensile strength $\frac{\sigma_{c}}{\sigma_{t}}=8$, where $\sigma_{c}$ is the uniaxial compressive strength. Using test data from one uniaxial and triaxial compression tests, the failure strength $\sigma_{f}$ can be predicted using this criterion, given by Equation (16). The Griffith criterion underpredicts the failure strength for 60 data points, which represents 90% of the dataset as shown in Figure 3c. The poor performance of the Griffith criterion in predicting $\sigma_{f}$ is attributed to the limitation of the failure envelope curvature coefficient (*n*) to ½, which depends on the mechanical properties of the rock [44]. The application of this criterion requires the measurement of the UCS of the rock while the proposed model does not require the measurement of the UCS. However, Griffith’s criterion yields the second-best estimate of $\sigma_{f}$ with an RMSE value of 42.11.

### 3.4. Renshaw and Schulson Criterion

**Figure 5:**
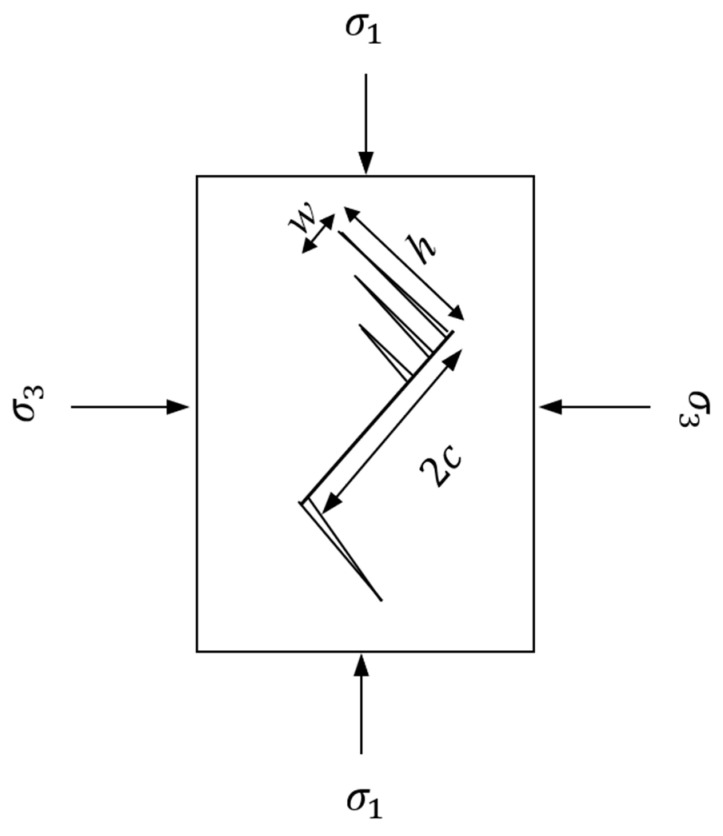
Microcracks emanating from pre-existing flaws.

Renshaw and Schulson [7] proposed a failure criterion based on the brittle behavior of crystalline materials such as ice and rocks. The criterion is based on the concept of secondary cracks emanating from primary cracks creating a parallel set of microcolumns. Considering a primary crack of length 2*c* inclined at 45° to the major principal stress direction ($\sigma_{1}$), as shown in Figure 5, the length of secondary cracks is h with the spacing between them referred to as w. The slenderness ratio of the microcolumns is governed by the length and spacing of microcolumns and is equal to α=h/w. Schulson et al. [17] have shown in experimental observations conducted on ice (which is used as a model material for rocks) that the slenderness ratio α ranges between 3.1 and 7.2; therefore, an average α value of 5.2 is adopted in this study.

The onset of failure is triggered by the bending-induced failure of these microcolumns. This bending and failure of microcolumns happen due to sliding along the primary crack, which is like the break of teeth in a comb under a sliding thumb. The frictional sliding along the primary crack will occur if the confinement ratio $\lambda =\frac{\sigma_{3}}{\sigma_{1}}<\frac{\left(1-\mu \right)}{\left(1+\mu \right)}$, since higher confinement suppresses the frictional sliding and leads to ductile failure. The failure strength $\sigma_{f}$ using this criterion is predicted as follows:

(17) $$\frac{\sigma_{f}\sqrt{c}}{K_{\mathrm{Ic}}}=\frac{2}{\left\{\sqrt{\sqrt{1+{\left(1-\mu \;\frac{\left(1+\lambda \right)}{\left(1-\lambda \right)}\right)}^{2/3}}-1\;}\right\}\left\{\sqrt{1+3{\;\mu }^{2}\alpha^{2}{\left(1-\lambda \right)}^{2}}\right\}}$$

Renshaw and Schulson [7] have adopted a value of 1 mm for 2*c* and a value of 1 $MPa\sqrt{m}$ for the $K_{Ic\;}$of sandstone. Substituting the value of μ=0.5 based on an experimental study conducted by Barnes et al. [45], where they reported that the μ for ice under −10 °C and sliding velocity of around 10^−5^ m/s and substituting α=5.2, the $\sigma_{f}$ can be predicted for different confinements. The scatteredness of the results shown in Figure 3d is attributed to the approximation adopted for the criterion parameters, such as the slenderness ratio of the microcolumns, and the μ, since this criterion was developed originally for ice, which slightly differs in nature from rocks. The RMSE and MAD values using Renshaw and Schulson criteria were the highest among the other failure criteria in this study with 69.07 and 56.41 for RMSE and MAD, respectively.

### 3.5. Modified Wiebols and Cook Criterion

Zhou [46] proposed a failure criterion which is an extension of the circumscribed Drucker–Prager criterion with features similar to the criterion of Wiebols and Cook [47]. This criterion describes the sliding microcrack surfaces that cause failure when the stress condition meets the frictional criterion. The failure criterion proposed by Zhou predicts rock failure strength when

(18) $$J_{2}^{1/2}=A+B\;J_{1}+C\;J_{1}^{2}$$

where $J_{1}$ is the mean effective confining pressure and equals $\frac{1}{3}\times \left(\sigma_{1}+\sigma_{2}+\sigma_{3}\right)$, and $J_{2}^{1/2}=\sqrt{\frac{1}{6}{\left(\sigma_{1}-\sigma_{2}\right)}^{2}+{\left(\sigma_{1}-\sigma_{3}\right)}^{2}+{\left(\sigma_{2}-\sigma_{3}\right)}^{2}}$. The parameters *A*, *B* and *C* are determined by Equations (19), (20) and (21), respectively, such that Equation (18) is constrained by rock strengths under biaxial ${(\sigma }_{1}=\sigma_{2})$ and triaxial conditions ${(\sigma }_{2}=\sigma_{3})$.

(19) $$A=\frac{C_{o}}{\sqrt{3}}-\frac{C_{o}}{3}\;B-\frac{{C_{o}}^{2}}{9}\;C$$

(20) $$C=\frac{\sqrt{27}}{2\;C_{1}+\left(q-1\right)\sigma_{3}-C_{o}}\times \left(\frac{C_{1}+\left(q-1\right){\;\sigma }_{3}-C_{o}}{{2C}_{1}+2\left(q+1\right){\;\sigma }_{3}-C_{o}}-\frac{q-1}{q+2}\right)$$

(21) $$B=\frac{\sqrt{3}\;(q-1)}{q+2}-\frac{C}{3}\;(2{\;C}_{o}+\left(q+2\right)\sigma_{3})$$

where $C_{o}$ is the UCS of the rock, $q={tan}^{2}\left(\frac{\pi }{4}+\varphi /2\right),$ and $C_{1}=\left(1+0.6\mu_{o}\right)C_{o}$.

The predicted strength using Wiebols and Cook criterion was determined for each rock under triaxial condition ${(\sigma }_{2}=\sigma_{3})$, by first calculating the constants *A*, *B* and *C* using Equations (19), (20) and (21), respectively. Knowing the measured UCS for each rock, the $J_{2}^{1/2}$ is calculated using Equation (18), through which $\sigma_{f}$ can be estimated. The estimated strength using the Wiebols and Cook criterion was calculated based on the measured friction angle and UCS; however, our proposed model does not require UC tests for the determination of UCS and predicting the failure strength. Despite this, it still resulted in a lower RMSE and slightly higher MAD values of the predicted strength, which better validates our proposed model.

The modified Wiebols and Cook criterion tends to overpredict the failure strength as shown in Figure 3e. The fitting of this criterion is good for a low range of $\sigma_{3}\;$(i.e., $\sigma_{3}$~4–10 MPa), while others present a poor fit due to the varying dependence of the intermediate stress $\sigma_{2}\;$under different $\sigma_{3}$. However, this criterion is the third best fit of experimental data after our proposed model and Griffith criterion based on the RMSE and MAD values of 43.62 and 31.39, respectively.

## 4. Parametric Study

**Figure 6:**
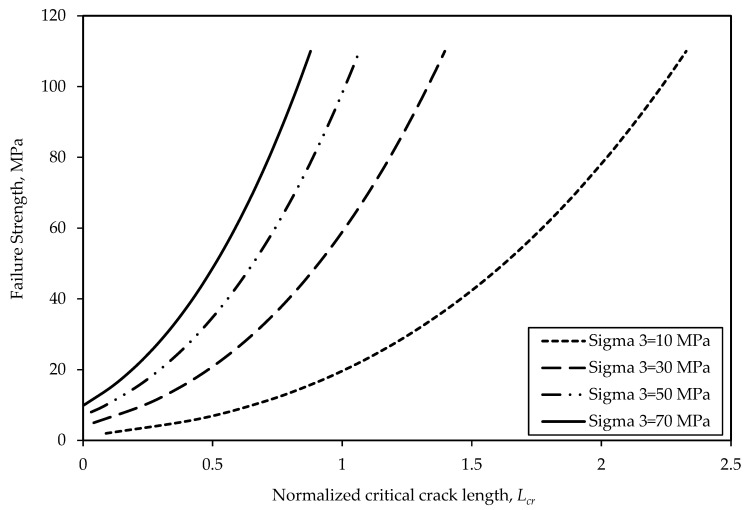
Relationship between the failure strength and normalized critical crack length.

Under triaxial compression with moderate confining pressure, local tensile stresses develop leading to microcracks generation [48]. However, under higher confinement, stress variations are within a purely compressive stress field. Therefore, higher confining pressure suppresses the growth and interaction of cracks, as shown in Figure 6, where the $L_{cr}$ decreases as the confining pressure increases. For example, when the confining pressure increases from 10 to 50 MPa, the normalized critical crack length decreases from 2.02 to 0.89, indicating a 56% decrease. The failure strength increases as the confining pressure increases due to the strengthening effect of confinement on the rock strength.

**Figure 7:**
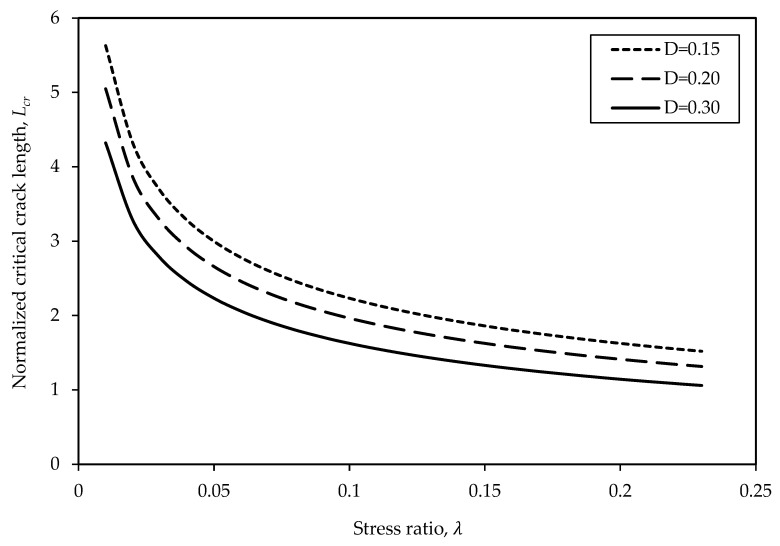
Relationship between the normalized critical crack length and stress ratio under constant damage levels.

Referring to Equation (6), two parameters that influence the *L_cr_* are the λ and *D*. To determine the qualitative effect of λ, the initial constant *D* is assumed for a given rock type in this study. Figure 7 shows that the *L_cr_* decreases as the λ increases and decreases with the increase in *D* at the same λ. In other words, the failure strength of the rock increases with the increase in the *L_cr_*. In addition, increasing the λ leads to an increase in the failure strength. However, the increase in *D* decreases the failure strength.

For example, at a constant *D* of 0.15, the *L_cr_* decreases from 2.23 to 1.63 when λ increases from 0.1 to 0.2, indicating a 27% decrease in the *L_cr_*. On the other hand, at a constant λ of 0.1, the *L_cr_* decreases from 1.63 to 1.14 when the initial *D* increases from 0.15 to 0.30, indicating a 30% decrease in the *L_cr_*, and therefore, a decrease in the failure strength. This finding is consistent with a study by Zhang et al. [20] where they proved that the confining pressure has a significant effect on long-wing cracks based on polyaxial and true triaxial compression tests conducted on jointed rock masses. They indicated that it is hard to form long-wing cracks under high confinement because tensile fractures are suppressed under high confining pressure compression.

## 5. Conclusions

The present study investigated the mechanical and fracture behavior of sandstone under triaxial compression. A new analytical model was proposed to determine the normalized critical crack length ($L_{cr}$), through which the failure strength can be estimated based on fracture mechanics applied to wing cracks emanating from the tips of pre-existing cracks. Many earlier studies have focused on conducting expensive and time-consuming laboratory tests to capture the mechanical and fracture behavior of rocks, while other studies focused on the uniaxial compression condition only ignoring the effect of confinement on the mechanical behavior of rocks. Therefore, this study was motivated by the need to develop a reliable analytical model to describe the mechanical behavior of rocks under triaxial compression using easily measured parameters. However, it is important to note that the proposed wing crack model is mostly applicable to rocks with Ψ angles less than 30° that guarantees a tension failure mode. The main findings drawn from this study are as follows:

1. The proposed model describes that the $L_{cr}$ decreases as the λ and *D* of the rock increase since higher confinements and higher levels of damage suppress the growth of cracks. The parametric study indicated that at a constant *D* of 0.15, the *L_cr_* decreases from 2.23 to 1.63 when λ increases from 0.1 to 0.2, indicating a 27% decrease in the *L_cr_*. On the other hand, at a constant λ of 0.1, the *L_cr_* decreases from 1.63 to 1.14 when the *D* increases from 0.15 to 0.30, indicating a 30% decrease in the *L_cr_*, and therefore, a decrease in the failure strength;

2. The proposed critical crack length is employed to predict the failure strength of sandstone. The total stress intensity is set equal to the rock $K_{IC}$ and the normalized crack length is set equal to the $L_{cr}$; therefore, the corresponding strength represents the predicted rock failure strength. The proposed model describes that the failure strength increases as the λ and $K_{IC}$ of the rock increases and decreases with the increase in *D* and the length of the pre-existing crack;

3. The proposed model assumes a value of 1 mm, 1.27 $MPa\sqrt{m}$, and 0.15 for the *a*, $K_{IC}$, and *D* for sandstone. The only unknown is the confining pressure. Therefore, no laboratory tests are required to determine the model parameters and predict the $\sigma_{f}$;

4. The proposed model was compared with widely accepted failure criteria to determine which parameters best describe the behavior of sandstone by minimizing the difference between the predicted and measured failure strength. The comparative study showed that our proposed model provides a better prediction of compressive strength based on the lowest RMSE and MAD values;

5. The HB and the modified Weibols and Cook criteria overpredict the failure strength, the Griffith criterion underpredicted the strength of 90% of the sandstones, and the Renshaw and Schulson criterion yields the greatest scatteredness of the strength prediction with the highest RMSE and MAD of 69.07 and 56.41, respectively.

It is important to note that the State of Wyoming, WYDOT, and the University of Wyoming hold a royalty-free, nonexclusive, unlimited, and irrevocable license to reproduce, publish, or otherwise use, and authorize others to use the copyright in any work that is generated from the research project entitled “Improving Design and Construction of Transportation Infrastructure through Bedrock Characterization”.

## Figures and Tables

**Table 1:** Summary of triaxial compression test results of sandstones from Wyoming.

Sample ID	Formation	Geological Age	Depth m	*n* ^1^ %	$\sigma_{3}$ ^2^ MPa	$\sigma_{f}$ ^3^ MPa	*c*^4^ MPa	φ ^5^ Degree
16	Flathead	Cambrian	4.76	2.20	4	95.59	20.34	37
			5.85	3.50	8	120.12		
19	Aspen	Cretaceous	6.10	3.43	10	98.89	5.38	49
23	Lance	Cretaceous	Surface	12.18	4	36.50	11.00	8
31	Tensleep	Pennsylvanian	Surface	13.20	4	59.88	13.10	48
32	Arikaree	Lower Miocene	Surface	11.70	4	45.86	2.76	51
33	Arikaree	Lower Miocene	Surface	12.20	4	46.49	3.10	54
				11.10	10	111.03		
39	Hanna	Paleocene	44.15	13.10	4	33.19	2.48	46
			44.60	15.20	10	70.72		
41	Hanna	Paleocene	23.32	14.80	4	31.47	2.34	44
			24.09	16.80	6	42.96		
43	Wind River	Eocene	Surface	14.50	4	14.00	8.96	56
				14.00	10	21.11		
49	Bridger	Eocene	Surface	27.20	4	34.68	4.48	29
				24.20	10	42.44		
50	Fort Union	Paleocene	Surface	12.20	4	31.48	1.38	47
51	Fort Union	Paleocene	Surface	3.92	4	143.53	5.52	55
				5.92	10	53.79		
56	Casper	Permian	Surface	11.10	4	72.56	7.93	53
				10.20	10	132.23		

1 Porosity in percentage, 2 confining pressure in MPa, 3 failure strength in MPa, 4 cohesion in MPa, 5 internal friction angle in degree.

**Table 2:** Summary of triaxial compression test results of sandstones from the literature.

Formation (Location)	*n* ^1^ %	*σ*_3_ ^2^ MPa	$\sigma_{f}$ ^3^ MPa	*c* ^4^ MPa	φ ^5^ Degree	Reference
Vosges, (France)	22.00	10–60	83.00–178.00	21.50	35.00	[32]
Red sandstone, (China)	8.90	5–35	115.10–236.10	22.42	37.80	[2]
Hawkesbury, (Australia)	13.00	4–25	58.00–116.00	16.50	28.00	[33]
Gosford, (Australia)	16.00	10–30	109.90–172.40	22.60	31.50	[34]
Jurassic sandstone	-	3–7	84.00–169.00	2.96	63.89	[35]
Black sandstone, (NA) ^6^	1.50	10–60	131.86–241.81	22.50	31.97	[36]
Red sandstone, (NA) ^6^	2.00	10–60	93.40–131.49	13.00	26.25	
-	-	5–25	95.50–220.50	-	67.50	[37]
-	8.70	5–25	116.50–202.00	26.40	36.47	[38]
-	16.24	5–15	105.50–126.00	33.10	21.10	[39]

1 Porosity in percentage, 2 confining pressure in MPa, 3 compressive strength in MPa, 4 cohesion in MPa, 5 internal friction angle in degree, 6 not Available.

## Data Availability

The original contributions presented in this study are included in the article. Further inquiries can be directed to the corresponding author.

## List of Abbreviations

μ	Coefficient of friction
c	Cohesion
$\sigma_{3}$	Confining pressure
*Ψ*	Crack inclination angle
l	Crack length
$\sigma_{f}$	Failure compressive strength
a	Half-length of the preexisting crack
D	Initial level of damage
φ	Internal friction angle
$\sigma_{1}$	Major principal stress
$m_{i}$	Material constant for intact rock
$J_{1}$	Mean effective confining pressure
$L_{cr}$	Normalized critical crack length
$N_{A}$	Number of initial cracks per unit area
n	Porosity
$K_{I}$	Stress intensity factor due to crack initiation
$K_{I}^{I}$	Stress intensity factor due to crack interaction
*λ*	Stress ratio
$\sigma_{ci}$	Rock uniaxial compressive strength
$K_{IC}$	Type-I fracture toughness
$T_{o}$	Uniaxial tensile strength
